# Extra-renal disease patterns in lupus nephritis: clinical and serological activity confers increased risk of renal flares and damage accrual

**DOI:** 10.1093/rheumatology/keag604

**Published:** 2026-10-27

**Authors:** Konstantinos Drougkas, Spyridon Katechis, Myrto Nikoloudaki, Myrto Kostopoulou, Christina Tsalapaki, Sofia Flouda, Maria Pappa, Marija Bakula, Ivan Padjen, Pinelopi Konstantopoulou, George Moustakas, Aggelos Banos, Dimitrios Vassilopoulos, George Bertsias, Paraskevi V Voulgari, Dimitrios T Boumpas, Antonis Fanouriakis

**Affiliations:** Rheumatology and Clinical Immunology Unit, Attikon University Hospital, National and Kapodistrian University of Athens Medical School, Athens, Greece; Rheumatology and Clinical Immunology Unit, Attikon University Hospital, National and Kapodistrian University of Athens Medical School, Athens, Greece; Rheumatology, Clinical Immunology and Allergy Department, University Hospital of Heraklion, University of Crete, Heraklion, Greece; Department of Nephrology, “G. Gennimatas” General Hospital, Athens, Greece; Clinical Immunology—Rheumatology Unit, 2nd Department of Medicine and Laboratory, National and Kapodistrian University of Athens, General Hospital of Athens Hippokration, Athens, Greece; Rheumatology and Clinical Immunology Unit, Attikon University Hospital, National and Kapodistrian University of Athens Medical School, Athens, Greece; Rheumatology and Clinical Immunology Unit, Attikon University Hospital, National and Kapodistrian University of Athens Medical School, Athens, Greece; University of Zagreb, School of Medicine, Division of Clinical Immunology and Rheumatology, Croatian Referral Centre for SLE and Related Diseases, Department of Internal Medicine, University Hospital Centre Zagreb, Zagreb, Croatia; University of Zagreb, School of Medicine, Division of Clinical Immunology and Rheumatology, Croatian Referral Centre for SLE and Related Diseases, Department of Internal Medicine, University Hospital Centre Zagreb, Zagreb, Croatia; Department of Rheumatology, “G. Gennimatas” General Hospital, Athens, Greece; Department of Nephrology, “G. Gennimatas” General Hospital, Athens, Greece; Rheumatology and Clinical Immunology Unit, Attikon University Hospital, National and Kapodistrian University of Athens Medical School, Athens, Greece; Clinical Immunology—Rheumatology Unit, 2nd Department of Medicine and Laboratory, National and Kapodistrian University of Athens, General Hospital of Athens Hippokration, Athens, Greece; Rheumatology, Clinical Immunology and Allergy Department, University Hospital of Heraklion, University of Crete, Heraklion, Greece; Department of Rheumatology, School of Health Sciences, Faculty of Medicine, University of Ioannina, Ioannina, Greece; Rheumatology and Clinical Immunology Unit, Attikon University Hospital, National and Kapodistrian University of Athens Medical School, Athens, Greece; Rheumatology and Clinical Immunology Unit, Attikon University Hospital, National and Kapodistrian University of Athens Medical School, Athens, Greece

**Keywords:** lupus nephritis, extra-renal disease activity, anti-dsDNA antibodies, latent class analysis

## Abstract

**Objective:**

The objective of this study was to explore patterns of extra-renal disease activity following renal response in LN and evaluate their association with renal flares and organ damage accrual.

**Methods:**

This is a multicentre retrospective cohort study of patients with biopsy-proven LN who have achieved renal response. Latent class analysis (LCA) was applied to identify patterns of extra-renal activity. Associations with adverse outcomes were examined using Cox proportional hazards models.

**Results:**

A total of 168 patients were included. Over a median follow-up of 4.0 years, 92.3% of patients exhibited extra-renal activity at least once. Serological activity (anti-dsDNA positivity and/or hypocomplementemia) was the most frequent active domain, followed by mucocutaneous and musculoskeletal involvement. Clinically significant extra-renal flares occurred in 37.5% of patients. Higher non-renal SLEDAI-2K scores were independently associated with increased risk of subsequent renal flares [adjusted hazard ratio (aHR) 1.18, 95% CI 1.07–1.30] and damage accrual (aHR 1.23, 95% CI 1.13–1.35), while BILAG-A extra-renal flares were strongly associated with damage accrual (aHR 10.56, 95% CI 4.63–24.06). LCA identified three classes of extra-renal activity: (i) mucocutaneous/musculoskeletal, (ii) combined serological–clinical activity, and (iii) predominant hypocomplementemia. Only the combined phenotype was independently associated with renal flares (aHR 2.66, 95% CI 1.34–5.30) and damage accrual (aHR 2.37, 95% CI 1.07–5.25). In exploratory analyses stratified by anti-dsDNA status, clinical extra-renal activity was associated with adverse outcomes among anti-dsDNA–positive visits.

**Conclusion:**

Extra-renal activity in LN following renal response is common. Anti-dsDNA positivity is the main factor associated with adverse outcomes, while concomitant clinical extra-renal activity may further increase risk among serologically active patients.

Rheumatology key messages

- Extra-renal activity following renal response is common and independently predicts renal flares and damage accrual.
- Three distinct extra-renal activity patterns exist, but only the combination of anti-dsDNA positivity with clinical extra-renal activity is associated with adverse outcomes.
- Clinical extra-renal activity adds prognostic value mainly in anti-dsDNA–positive patients.

## Introduction

LN remains a major contributor to morbidity and mortality in patients with SLE [1]. Up to 50% of patients may develop chronic kidney disease (CKD) during the disease course [2], with a non-negligible proportion also progressing to end-stage kidney disease (ESKD) [3]. The main driver of irreversible kidney damage accrual is the occurrence of renal flares, which leads to progressive loss of nephrons and kidney function decline [4].

The clinical significance of residual activity in LN has been extensively studied, and current treatment targets focus both on the resolution of proteinuria and the stabilization of kidney function [5]. However, even among patients who achieve complete renal response, a substantial proportion experience disease relapses and consequent damage accrual [6]. Long-term benefits of sustained renal response have been shown [7], yet the contribution of residual non-renal activity on the risk for flares has received little attention to date. We recently showed that early and sustained attainment of systemic disease control following renal response confers protection against renal flares and accrual of organ damage in patients with LN [8]. A significant impact of extra-renal symptoms on the quality of life of patients with LN has also been recently described [9]. Lastly, the prognostic value of longitudinal assessments of serological markers, a component of non-renal activity, has long been debated [10], with most studies underscoring the independent importance of anti-dsDNA positivity [11, 12]. Nevertheless, a systematic evaluation of extra-renal disease activity patterns during the maintenance phase of LN treatment, and their relationship with hard clinical outcomes, has not been performed.

Based on the above, herein we sought (i) to provide a comprehensive description of extra-renal disease activity in patients with LN following renal response, (ii) to identify distinct activity patterns using a data-driven approach, and (iii) to evaluate their association with renal flares and organ damage accrual.

## Methods

### Study population

We performed a multicentre retrospective cohort study in adult SLE patients (classified according to the 2012 SLICC [13] and/or 2019 EULAR/ACR [14] criteria), with a first diagnosis of biopsy-proven LN (2003 ISN/RPS class II, III, IV, V, or mixed [15]) who were followed at seven referral centres between January 2010 and December 2025. Eligibility for the study required (i) achievement of partial (PRR) or complete renal response (CRR) following treatment for LN, and (ii) a minimum follow-up duration of ≥3 months. Further, the minimum required visits were every 3 months during the first year, and every 6 months afterwards.

### Data collection & definitions

The enrolment date for each patient was defined as the date of first renal response. CRR was defined as a urine protein:creatinine ratio (UPCR) of <50 mg/mmol (or proteinuria of <0.5 g/24 h) with normal or near-normal estimated glomerular filtration rate (eGFR; within 10% of baseline if previously abnormal); PRR was defined as a ≥50% reduction in proteinuria to subnephrotic levels with normal or near-normal eGFR [16].

Baseline and follow-up data were extracted from hospital medical records. At the enrolment visit, the following were documented: demographics (age, sex), disease history (dates of SLE and LN diagnosis, prior major SLE manifestations and treatments), kidney disease features (histological features, type of response, kidney function parameters), and immunological profile (anti-dsDNA, complement levels). Anti-dsDNA antibodies were measured using commercially available ELISA assays, with positivity defined according to the respective laboratory reference range. For this study, the components of the SLEDAI 2000 (SLEDAI-2K) [17] were captured at each visit, as well as the occurrence of renal and extra-renal flares, and the development of damage accrual events (for definitions, see Supplementary Data S1).

Extra-renal disease activity at each visit was assessed with the SLEDAI-2K, excluding the four renal components (urinary casts, haematuria, pyuria, and proteinuria). The remaining SLEDAI-2K components were grouped into eight domains: neurological (seizure, psychosis, organic brain syndrome, visual disturbance, cranial nerve disorder, lupus headache, and cerebrovascular accident), vascular (vasculitis), musculoskeletal (arthritis and myositis), mucocutaneous (rash, alopecia, and mucosal ulcers), serosal (pleurisy and pericarditis), serological (low complement and anti-dsDNA), constitutional (fever), and haematological (thrombocytopenia and leukopenia). Clinical extra-renal activity included all active non-renal SLEDAI-2K components, except the serological. Extra-renal disease activity was also assessed using the BILAG-2004 index [18], with extra-renal flares defined as new-onset BILAG-A or BILAG-B activity in any non-renal domain.

### Statistical analysis

Descriptive statistics are presented in Supplementary Data S1.

The association of extra-renal disease activity with renal flares and damage accrual was examined using Cox proportional hazards models with the Anderson–Gill counting process method. Robust variance estimation was applied through clustering by patient to account for within-patient correlation. Extra-renal disease activity was assessed by: (i) the non-renal SLEDAI-2K, measured as a continuous time-varying predictor (per unit increase), and (ii) the occurrence of BILAG extra-renal flares (any flare, A flare, B flare). Time-varying extra-renal activity measures were lagged by one visit to ensure temporality, such that extra-renal activity preceded the renal flare or damage accrual event.

Latent class analysis (LCA) was applied to visits with at least one active non-renal SLEDAI-2K component. Visits with no active non-renal SLEDAI-2K component were defined as ‘extra-renal quiescent’ and excluded from the LCA. LCA was applied to binary indicators of individual non-renal SLEDAI-2K components, while components with zero variance were excluded. The optimal number of classes was selected based on the Bayesian Information Criterion (BIC), with lower values indicating better model fit penalized for model complexity. Visits were assigned to their most probable class via modal assignment. The resulting latent classes were then combined with the extra-renal quiescent state to produce a unified time-varying disease state variable. This time-varying disease state was subsequently used as a predictor in Anderson–Gill Cox models with the extra-renal quiescent state as the reference category.

All Cox models were fitted as unadjusted and adjusted for age, sex, type of initial renal response, baseline SDI, proliferative LN histology, SLE duration before LN, and glucocorticoid dose as a time-varying covariate. To confirm the validity of the findings, sensitivity analyses were performed by further adjusting the main models for time-varying MMF use and, separately, time-varying belimumab use. Additional sensitivity analyses were performed by restricting the cohort to patients with at least 12 months of follow-up and, separately, to patients who achieved complete renal response at inclusion; the latter models were adjusted for age and sex only to avoid overfitting. Lastly, stratified analyses examined the associations of clinical non-renal SLEDAI-2K and low complement levels with renal flares and damage accrual, according to anti-dsDNA status. To avoid overfitting due to the smaller number of events within strata, these stratified models were adjusted for age, sex, and type of initial renal response. All analyses were performed with R (R Foundation for Statistical Computing, Vienna, Austria, version 4.5.1).

## Results

### Cohort characteristics and patterns of extra-renal disease activity

A total of 168 patients with LN who achieved renal response were followed for a median [interquartile range (IQR)] of 4.0 (4.6) years (total 1933 follow-up visits). Patient characteristics at inclusion are presented in Table 1 and distribution across participating centres in Supplementary Table S1. The mean (s.d.) age at enrolment was 38.7 (14.6) years, and proliferative LN histology was present in 72.0% of patients. LN was present at SLE diagnosis in 74 patients (44%), while 37 (22%) had at least one major organ manifestation before LN. At baseline (i.e. enrolment visit), CRR was reached in 108 patients (64.3%), while the remaining 60 (35.7%) achieved PRR, with a median (IQR) time to first renal response of 5.0 (5.0) months. The median prednisone dose decreased from 10 mg/day at inclusion to 5 mg/day across follow-up visits. During follow-up, MMF was the most frequent treatment, observed in 63.2% of visits and received by 80.4% of patients at least once, followed by belimumab and AZA.

**Table 1:** Cohort characteristics at inclusion and during follow-up.

Characteristic	Value
**At inclusion visit**	
Age, years, mean (s.d.)	38.7 (14.6)
Female sex, *n* (%)	140 (83.3)
Disease duration, years, median (IQR)	1.9 (6.8)
Major organ manifestations prior to LN, *n* (%)	
- Neuropsychiatric	10 (6)
- Haematological	21 (12.5)
- Cardiorespiratory	12 (7.1)
- Gastrointestinal	1 (0.6)
Proliferative histology (class III/IV ± V), *n* (%)	121 (72.0)
NIH activity index, median (IQR)	8 (6)
NIH chronicity index, median (IQR)	3 (3)
Complete renal response, *n* (%)	108 (64.3)
Time to first renal response, months, median (IQR)	5.0 (5.0)
SLEDAI-2K, median (IQR)	4 (6)
Non-renal SLEDAI-2K, median (IQR)	2 (3)
PGA, mean (s.d.)	1.1 (0.5)
SDI >0, *n* (%)	54 (32.1)
Proteinuria, g/day, median (IQR)	0.4 (0.5)
Haematuria, *n* (%)	27 (16.1)
eGFR, ml/min/1.73 m^2^, mean (s.d.)	94.2 (26.7)
Anti-dsDNA positive, *n* (%)	83 (49.4)
Low complement, *n* (%)	85 (50.6)
Prednisone dose, mg/day, median (IQR)	10 (10)
HCQ, *n* (%)	154 (91.7)
MMF, *n* (%)	89 (53.0)
AZA, *n* (%)	14 (8.3)
Belimumab, *n* (%)	26 (15.5)
Calcineurin inhibitor^a^, *n* (%)	8 (4.8)
**During follow-up**	
Number of visits, total	1933
Non-renal SLEDAI, median (IQR)	2 (4)
Visits with low complement, *n* (%)	726 (37.6)
Patients with low complement at least once, *n* (%)	101 (60.1)
Visits with anti-dsDNA positivity, *n* (%)	586 (30.3)
Patients with anti-dsDNA positivity at least once, *n* (%)	104 (61.9)
Visits with clinical extra-renal activity, *n* (%)	596 (30.8)
Patients with clinical extra-renal activity at least once, *n* (%)	108 (64.3)
Visits with any BILAG extra-renal flare, *n* (%)	137 (7.1)
Patients with any BILAG extra-renal flare at least once, *n* (%)	63 (37.5)
Prednisone dose across visits, mg/day, median (IQR)	5 (5)
Visits on MMF, *n* (%)	1221 (63.2)
Visits on AZA, *n* (%)	258 (13.3)
Visits on belimumab, *n* (%)	314 (16.2)
Visits on calcineurin inhibitor, *n* (%)	110 (5.7)

a Calcineurin inhibitors included voclosporin, tacrolimus, and CSA. eGFR: estimated glomerular filtration rate; IQR: interquartile range; NIH: National Institutes of Health; PGA: Physician Global Assessment; SDI: SLICC/ACR Damage Index; SLEDAI-2K: SLEDAI 2000.

At least one visit with non-renal SLEDAI-2K activity was recorded in 155 patients (92.3%), while clinical extra-renal activity (excluding serology) was observed in 108 patients (64.3%) at least once, and was present in 30.8% of all follow-up visits. Patients with major organ manifestations before LN onset exhibited higher non-renal activity during follow-up (Supplementary Fig. S1). Regarding specific domains, serological activity was the most commonly active domain during visits, with low complement and anti-dsDNA positivity recorded at 37.6% and 30.3% of visits, and affecting 60.1% and 61.9% of patients at least once during follow-up, respectively. Extra-renal flares (BILAG-A or BILAG-B) occurred in 37.5% of patients, accounting for 7.1% of all follow-up visits, with the mucocutaneous and musculoskeletal domains being the most frequently involved (Supplementary Fig. S2).

The various combinations of extra-renal activity during follow-up are shown in Fig. 1A. In 1208 visits with active extra-renal disease, isolated serological activity was the most common pattern (*n* = 612). Beyond serology, isolated mucocutaneous (*n* = 130, 10.8%) and isolated musculoskeletal involvement (*n* = 66, 5.5%) were the next most frequent single-domain patterns. Further, combinations of serologic activity with mucocutaneous (*n* = 165, 13.7%) or musculoskeletal (*n* = 50, 4.1%) domains represented the most prevalent multi-domain patterns, while haematological activity, though less frequent in isolation (*n* = 37, 3.1%), often appeared in combination with the serological and mucocutaneous domains.

**Figure 1:**
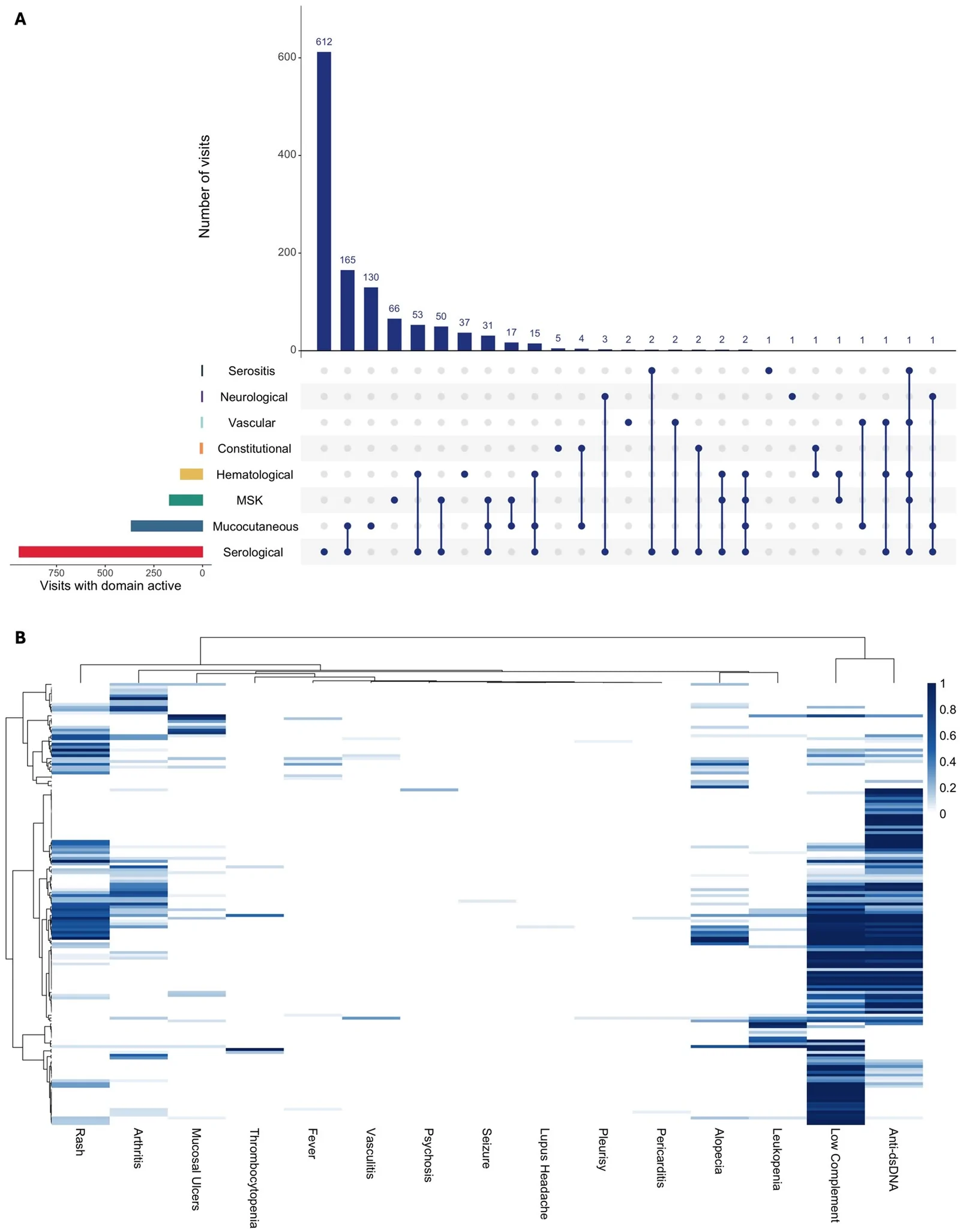
Patterns of extra-renal disease activity in LN patients following renal response. (A) UpSet plot displaying the frequency and concurrent presence of extra-renal SLEDAI-2K domains during follow-up visits. Horizontal bars (left) indicate the total number of visits in which each domain was active. Vertical bars represent the frequency of each combined pattern, ordered by decreasing frequency. Dots and connecting lines indicate which domains contribute to each intersection. (B) Heatmap displaying the proportion of follow-up visits during which each SLEDAI-2K extra-renal component was active, summarized per patient (rows). Values range from 0% (component never active) to 100% (component active at all visits). Only components with at least one active visit across the cohort are displayed. MSK: musculoskeletal; SLEDAI-2K: SLEDAI 2000.

At the patient level, extra-renal disease activity profiles varied considerably, with some patients showing persistent serological activity, others being characterized predominantly by mucocutaneous and musculoskeletal involvement, and others exhibiting more diffuse or intermittent patterns (Fig. 1B). Temporally, serological activity was most prevalent extra-renal activity early after renal response, affecting ∼65% of patients at baseline, and it declined gradually over follow-up, albeit with intermittent fluctuations, suggesting ongoing serological activity throughout the maintenance phase (Supplementary Fig. S3). All other domains remained consistently below 25% of patients at any given time point.

### Extra-renal disease activity increases the risk for renal flares and damage accrual

During follow-up, 59 renal flares occurred in 42 patients (25.0%), and 48 new organ damage events in 38 patients (22.6%). The association of extra-renal disease activity with renal flares and organ damage accrual is presented in Fig. 2. Each unit increase in the non-renal SLEDAI-2K score conferred an increased risk for both subsequent renal flares [adjusted hazard ratio (aHR) 1.18, 95% CI 1.07–1.30] and damage accrual (aHR 1.23, 95% CI 1.13–1.35) in multivariable analyses. Similar associations were observed when restricting the analysis to clinical non-renal activity excluding serological components, where each unit increase in clinical non-renal SLEDAI was independently associated with renal flares (aHR 1.15, 95% CI 1.01–1.30) and damage accrual (aHR 1.25, 95% CI 1.13–1.37).

**Figure 2:**
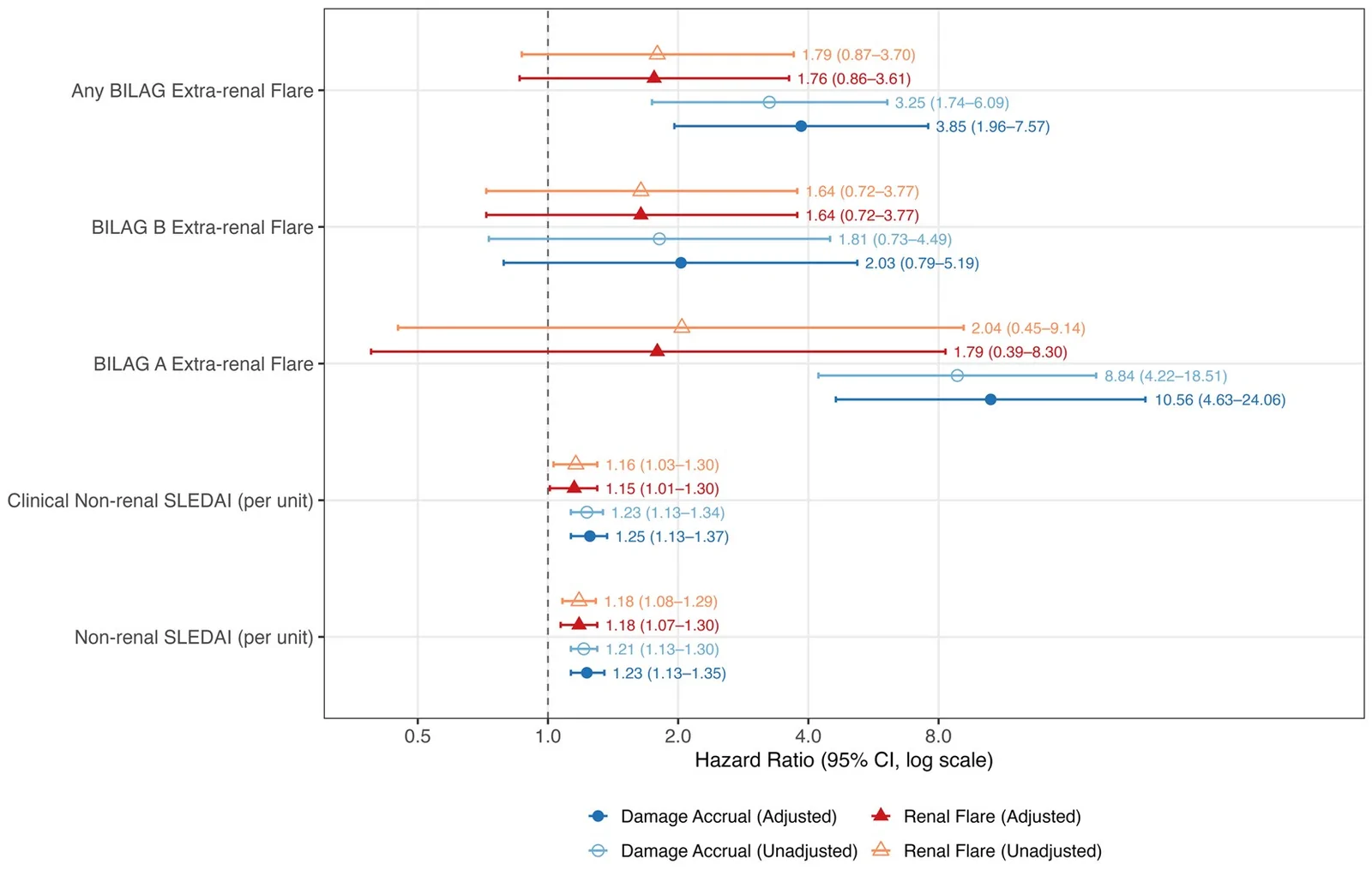
Association of extra-renal disease activity with renal flares and organ damage accrual. Forest plot with hazard ratios and 95% CIs for the association between extra-renal disease activity measures with renal flares and damage accrual events. Extra-renal activity was quantified as the non-renal SLEDAI-2K score and its clinical version excluding serologic components (both per unit increase) as well as the occurrence of BILAG extra-renal flares (grade A, grade B, or any grade) at each visit interval. All models used the Anderson–Gill counting process method to account for recurrent events, with robust variance estimation through patient clustering. Adjusted models (filled symbols) were adjusted for age, sex, type of initial renal response, baseline SDI, proliferative LN histology, SLE duration prior to LN, and glucocorticoid dose; unadjusted models are shown as open symbols for comparison. SDI: SLICC/ACR Damage Index; SLEDAI-2K: SLEDAI 2000.

A total of 137 extra-renal BILAG flares occurred in 63 (37.5%) patients, of which 32 were BILAG-A and 105 BILAG-B. The occurrence of any extra-renal BILAG flare was independently associated with subsequent organ damage accrual (aHR 3.85, 95% CI 1.96–7.57); association with renal flare risk, while directionally consistent, did not reach statistical significance. When stratified by flare severity, BILAG-A events emerged as the strongest predictor of subsequent damage accrual (aHR 10.56, 95% CI 4.63–24.06), representing the largest effect size observed for all predictors examined. In contrast, BILAG-B extra-renal flares showed only a trend toward increased risk for both renal flares and damage accrual, without reaching statistical significance for either outcome (Fig. 2). Results were consistent across sensitivity analyses additionally adjusting for MMF or belimumab use, and in analyses restricted to complete renal responders or patients with ≥12 months of follow-up (Supplementary Fig. S4).

### Combined clinical and serological extra-renal activity drives renal flares and damage accrual

To identify patterns of extra-renal activity, we applied LCA to visits with active extra-renal disease. The BIC curve showed a clear minimum at *k* = 3 classes, which was selected as the optimal solution; thus, three distinct activity classes were identified (Supplementary Fig. S5). Classification quality was excellent (entropy *R*^2^ = 0.92 and mean posterior probabilities 0.956, 1.000 and 0.904 for Classes 1, 2 and 3, respectively), indicating well-separated and reliably assigned latent classes (Supplementary Table S2).

The three identified extra-renal activity classes, along with their corresponding conditional item-response probabilities for the non-renal SLEDAI-2K components, are shown in Fig. 3. Class 1 (mucocutaneous/musculoskeletal; *n* = 315 visits, 26.1%) was characterized by moderate probability of musculoskeletal and mucocutaneous activity. Class 2 (serology/mucocutaneous/musculoskeletal; *n* = 586 visits, 48.5%) was defined by combined serological and clinical activity, with universal presence of anti-dsDNA positivity (probability = 1.00), together with mucocutaneous and musculoskeletal involvement, as well as low complement levels. Class 3 (hypocomplementemia; *n* = 307 visits, 25.4%) was characterized by universal hypocomplementemia (probability = 1.00), in the absence of anti-dsDNA positivity, and very low probabilities of rash, arthritis, and haematological involvement. The evolution of these classes during follow-up is shown in Supplementary Fig. S6. The serology/mucocutaneous/musculoskeletal class consistently represented the most prevalent active state, while visits in complete extra-renal quiescence increased proportionally after the median follow-up of 48 months (subsequent estimates should be interpreted with caution due to the decreasing number of patients). The distribution of maintenance treatments across these classes is shown in Supplementary Fig. S7, with MMF being the most frequent.

**Figure 3:**
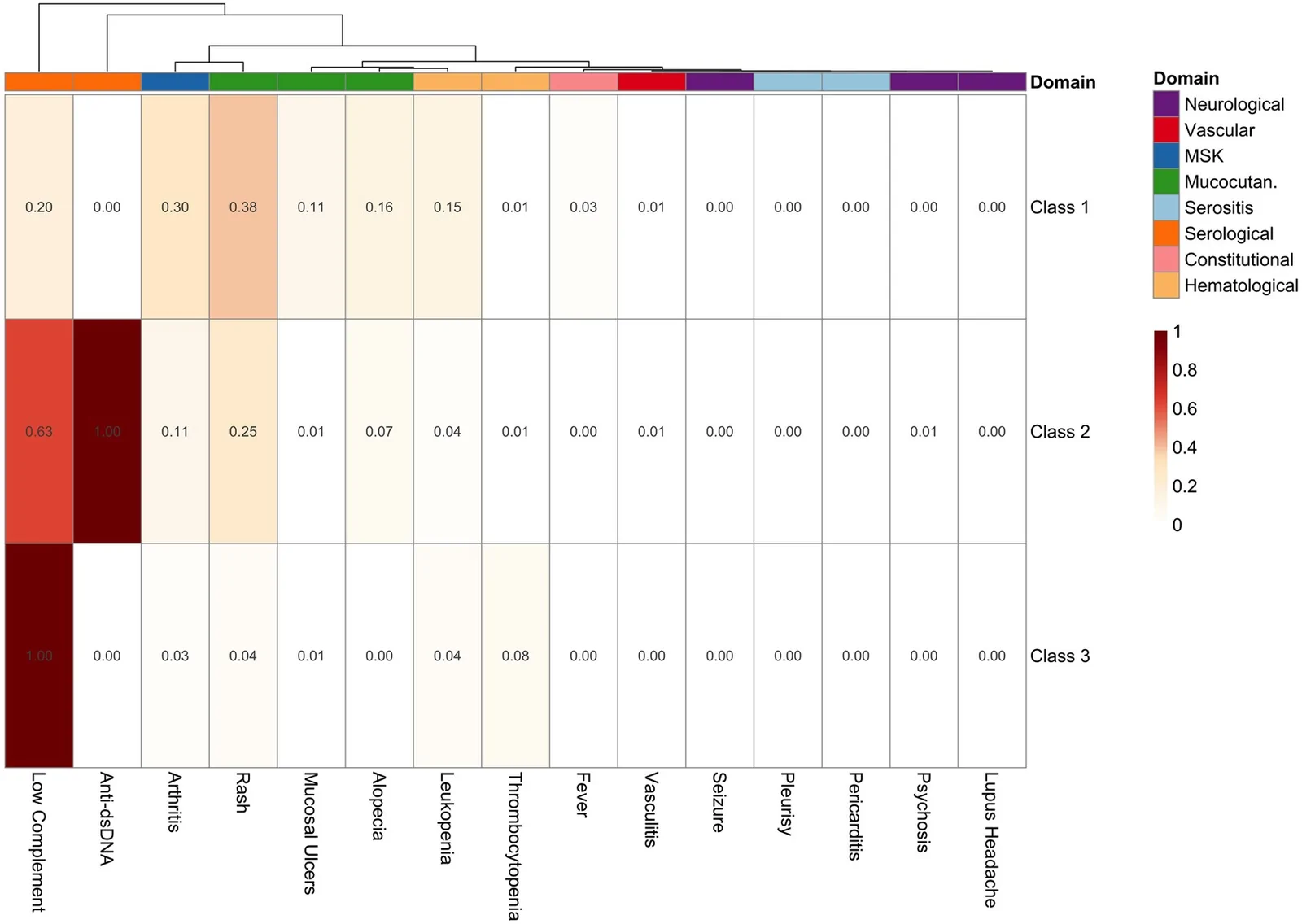
Conditional item-response probabilities defining the three latent classes of extra-renal disease activity in LN. Heatmap showing the item-response probabilities from a three-class latent class analysis model. Each cell represents the conditional probability that a given non-renal SLEDAI-2K component (columns) is active at a visit belonging to that latent class (rows). The three-class solution was selected based on the Bayesian Information Criterion (BIC). Class 1 is mainly characterized by musculoskeletal and mucocutaneous involvement. Class 2 is defined by combined serological and clinical extra-renal activity. Class 3 is characterized by universal hypocomplementemia, in the absence of anti-dsDNA positivity, and very low probabilities of rash, arthritis, and haematological involvement. MSK: musculoskeletal; Mucocutan.: mucocutaneous; SLEDAI-2K: SLEDAI 2000.

When these disease states were used as time-varying predictors with extra-renal quiescent visits as the reference, the serology/mucocutaneous/musculoskeletal class was independently associated with a more than 2-fold increased risk of renal flare (aHR 2.66, 95% CI 1.34–5.30) and damage accrual (aHR 2.37, 95% CI 1.07–5.25) (Fig. 4). In contrast, neither the mucocutaneous/musculoskeletal nor the hypocomplementemia class reached statistical significance for either outcome. These results remained directionally consistent across all sensitivity analyses performed (Supplementary Fig. S8). Of the 48 damage accrual events, 17 were renal, and 31 non-renal; their distribution across the disease states is shown in Supplementary Fig. S9.

**Figure 4:**
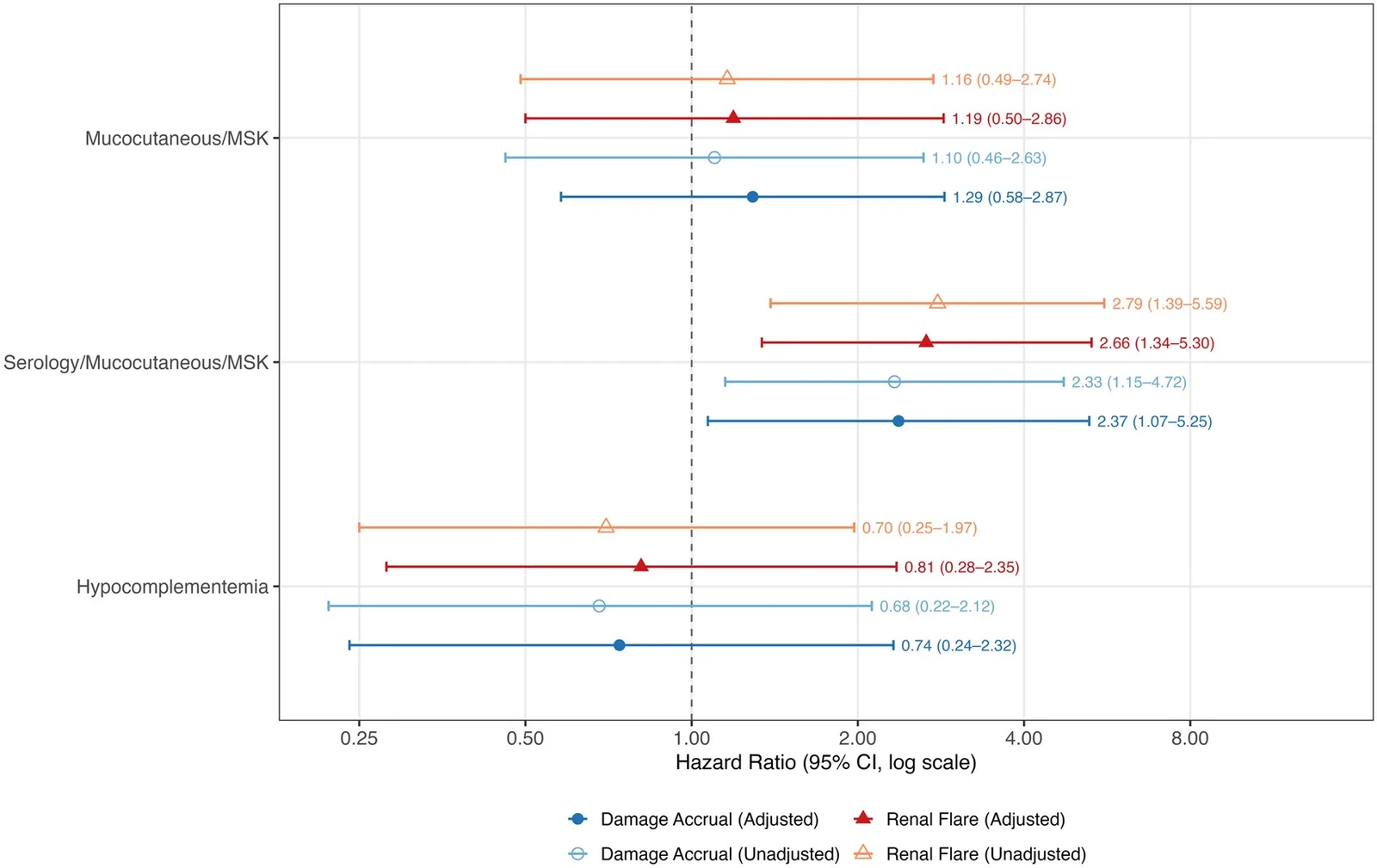
Association of three distinct extra-renal disease activity classes with renal flares and organ damage accrual. Forest plot with hazard ratios and 95% CIs for the association of extra-renal disease state with renal flares and damage accrual. Disease states were derived from latent class analysis where active visits were assigned to one of three latent classes: mucocutaneous/musculoskeletal (Class 1), serology/mucocutaneous/musculoskeletal (Class 2), and hypocomplementemia (Class 3). All models used the Anderson–Gill counting process method to account for recurrent events, with robust variance estimation through clustering by patient. The extra-renal quiescent state served as the reference category. Adjusted models (filled symbols) were adjusted for age, sex, type of initial renal response, baseline SDI, proliferative LN histology, SLE duration prior to LN, and glucocorticoid dose; unadjusted models are shown as open symbols for comparison. SDI: SLICC/ACR Damage Index. MSK: musculoskeletal.

### Association of clinical extra-renal activity with adverse outcomes according to anti-dsDNA status

We further explored whether the increased risk of adverse outcomes observed in Class 2 (serology/mucocutaneous/musculoskeletal) was driven solely by anti-dsDNA positivity, or was further augmented by clinical non-renal activity or low complement levels.

To this end, we examined the association of clinical non-renal SLEDAI and low complement levels with adverse outcomes, stratified by anti-dsDNA status (Table 2). We found that clinical non-renal SLEDAI was associated with renal flares among anti-dsDNA–positive visits (aHR 1.17 per unit, 95% CI 1.01–1.35), whereas no statistically significant association was observed among anti-dsDNA-negative visits (aHR 1.08, 95% CI 0.91–1.28). A similar pattern was observed for damage accrual, where clinical non-renal SLEDAI was associated with increased risk only in anti-dsDNA-positive visits (aHR 1.26, 95% CI 1.11–1.42), but not in anti-dsDNA-negative visits (aHR 1.08, 95% CI 0.90–1.31).

**Table 2:** Association of clinical non-renal SLEDAI and low complement with renal flare and damage accrual stratified by anti-dsDNA status.

Visits	Outcome	Clinical non-renal SLEDAI aHR (95% CI)	Low complement aHR (95% CI)
**All visits**	Renal flares	1.13 (1.01–1.28)	1.87 (1.03–3.41)
	Damage accrual	1.24 (1.13–1.35)	1.83 (1.00–3.34)
**Anti-dsDNA–negative**	Renal flares	1.08 (0.91–1.28)	1.12 (0.42–2.95)
	Damage accrual	1.08 (0.90–1.31)	1.07 (0.39–2.97)
**Anti-dsDNA–positive**	Renal flares	1.17 (1.01–1.35)	1.71 (0.81–3.59)
	Damage accrual	1.26 (1.11–1.42)	1.94 (0.85–4.45)

Adjusted hazard ratios (aHRs) with 95% CIs derived from Andersen–Gill models adjusted for age, sex, and baseline renal response. Clinical non-renal SLEDAI excludes serologic activity components (anti-dsDNA and complement).

Low complement levels were associated with increased risks of renal flare (aHR 1.87, 95% CI 1.03–3.41) and damage accrual (aHR 1.83, 95% CI 1.00–3.34) in the whole cohort. However, after stratification by anti-dsDNA status, these associations were attenuated and no longer statistically significant in both anti-dsDNA-positive and anti-dsDNA-negative visits.

## Discussion

Patterns of extra-renal activity and their relationship with long-term outcomes remain an underexplored dimension of LN. In a multicentre LN cohort, we showed that extra-renal disease activity during the post-response period is frequent, heterogeneous, and clinically relevant. A higher non-renal SLEDAI-2K was associated with renal flares and damage accrual, while the occurrence of BILAG-A extra-renal flares strongly predicted subsequent damage. We further identified three distinct activity patterns, of which only the class that combined anti-dsDNA positivity with low complement and clinical extra-renal activity was associated with adverse outcomes.

Extra-renal activity was highly prevalent following renal response in our study. In fact, >90% of patients had at least one visit with non-renal activity, while approximately one-third of visits showed clinical extra-renal activity. Hypocomplementemia and anti-dsDNA positivity represented the most commonly active domains. Beyond serology, mucocutaneous and musculoskeletal involvement were the predominant clinical manifestations, both in isolation and in combination with serology. Our findings corroborate data from the Accelerating Medicines Partnership RA/SLE Lupus Nephritis Network, in which 41% of patients with active LN had concurrent extra-renal manifestations [9]. Our data extend this observation longitudinally, demonstrating that extra-renal activity exists well beyond the acute phase of LN, with patterns evolving during follow-up. Serological activity was most prevalent early after renal response and gradually declined, while mucocutaneous and musculoskeletal involvement remained the predominant clinical manifestations throughout follow-up.

Our study also shows that extra-renal activity carries prognostic information. Each unit increase in non-renal SLEDAI-2K was associated with a higher risk of both renal flares and damage accrual. Similar associations were observed when serological components were excluded, indicating that prognostic significance was not entirely driven by anti-dsDNA and complements. The occurrence of any BILAG extra-renal flare (A or B) was also associated with an increased risk of damage accrual. For renal flares, neither BILAG-A nor BILAG-B extra-renal flare models reached statistical significance, although all estimates showed a consistent trend towards an increased risk. These associations should nevertheless be viewed in the context of real-world treatment changes: extra-renal flares may lead to immunosuppressive treatment intensification, which could subsequently affect the risk of adverse outcomes. To partly account for this, our models included time-varying glucocorticoid dose, and sensitivity analyses additionally adjusted for MMF and belimumab use, with consistent results. These findings complement our recent work, in which sustained attainment of low disease activity states after renal response was found to be associated with better outcomes [8]. A recent study also demonstrated that achieving both renal and extra-renal remission protects against impaired kidney function and flares [19]. Taken together, these data are in alignment with the 2025 EULAR recommendations for the management of LN, which reinforce the concept that LN is part of a systemic disease and should be managed with attention to both renal and extra-renal control [5].

We identified three distinct activity patterns by LCA analysis: a predominantly mucocutaneous/musculoskeletal class (Class 1), a dsDNA-positive dominant class, with frequent low complements and a variable burden of clinical extra-renal activity (Class 2), and a predominant hypocomplementemia class (Class 3). Importantly, only Class 2 was associated both with renal flares and damage accrual. Since all visits with anti-dsDNA positivity were assigned to this class, this result supports the concept that anti-dsDNA positivity is a cardinal prognostic feature in LN, consistent with prior literature [11, 12, 20–23]. At the same time, Class 2 was not purely serological: a subset of visits also exhibited clinical activity, as well as hypocomplementemia. Conversely, Class 1, which captured clinical extra-renal activity with minimal serological involvement, was not associated with adverse outcomes. Similarly, Class 3, characterized by predominant hypocomplementemia, was also not associated with renal flares or damage accrual. This led us to the important question of whether the excess risk associated with Class 2 is driven solely by anti-dsDNA positivity, or whether clinical activity contributes additional prognostic value. In stratified analyses, clinical non-renal SLEDAI was associated with renal flares and damage accrual among anti-dsDNA–positive visits, while the respective estimates among anti-dsDNA–negative visits did not reach statistical significance. The most balanced interpretation of these findings is that anti-dsDNA positivity is indeed the dominant risk factor mostly responsible for the prognostic value of non-renal lupus activity, while clinical manifestations may refine risk during serologically active visits; however, this interpretation is based on stratified analyses and should be considered exploratory.

Our findings regarding hypocomplementemia were more nuanced. Although low complement levels were overall associated with renal flares and damage accrual, these associations were no longer significant after stratification by anti-dsDNA status. One possible interpretation is that the prognostic effect of hypocomplementemia in the overall cohort is actually driven by anti-dsDNA positivity. A systematic review showed that anti-dsDNA positivity is more consistently associated with subsequent adverse outcomes (i.e. flares) than hypocomplementemia [10]. This said, in stratified analyses, effect estimates remained numerically higher among anti-dsDNA–positive visits than among anti-dsDNA–negative visits, although CIs were wide, limiting definitive conclusions.

Our study has limitations. SLEDAI-2K components were collected from medical records, which may have led to under-ascertainment of certain manifestations. Also, SLEDAI-2K does not capture all extra-renal manifestations included in BILAG, and some activity may therefore have been missed. The median follow-up of 4 years, with declining patient numbers after 60 months, limits the reliability of temporal trends at later time points. As with any LCA, the latent classes are statistical constructs that require validation in independent cohorts, and the assignment of visits to classes involves probabilistic uncertainty. Moreover, latent class assignment was performed at the visit level rather than the patient level; therefore, these classes should be interpreted as dynamic disease states rather than fixed phenotypes. LCA assumes local independence between indicators, and repeated visits from the same patient may introduce within-patient correlation, not fully accounted for during class estimation. HCQ use was near-universal, preventing adjustment for its potential confounding effect, which is relevant given its reported protective effect against flares in LN [24, 25]. Finally, the BILAG-A association with damage accrual, although statistically significant, was based on few events and had a wide CI, warranting cautious interpretation; similarly, the anti-dsDNA–stratified analyses were limited by modest event numbers, and should therefore be interpreted as exploratory, rather than definitive evidence of effect modification.

In conclusion, extra-renal disease activity is common during the post-response phase of LN, and is associated with adverse outcomes. Anti-dsDNA positivity is the major prognostic driver of renal flares and damage accrual, but clinical extra-renal activity may carry additional prognostic significance, particularly within anti-dsDNA–positive visits. These findings support a holistic approach to LN monitoring, wherein kidney parameters are interpreted alongside general disease activity.

The study was approved by the Ethics Committee of Attikon University Hospital (protocol number 46/20-01-2025). As the study relied on routinely collected and de-identified data, informed consent from patients was not required.

## Supplementary Material

keag604_Supplementary_Data

## Data Availability

Data are available upon reasonable request. The data that support the findings of this study are available from the corresponding author upon reasonable request.
