# Supplementary material for: Extra-renal disease patterns in lupus nephritis: clinical and serological activity confers increased risk of renal flares and damage accrual
Source: Rheumatology (Oxford). 2026 Oct 27;65(11):keag604. doi: 10.1093/rheumatology/keag604 (PMC13755872; doi:10.1093/rheumatology/keag604)
Supplement: Supplementary file 1 [file keag604_supplementary_data.docx]

**Supplementary Data S1: Methods**

*Definitions*

Renal flares were defined as proteinuric in case of a reproducible doubling of UPCR to >100 mg/mmol (or proteinuria >1 g/24h) after CRR, or to >200 mg/mmol (or proteinuria >2 g/24h) after PRR, or nephritic in case of reproducible increase in serum creatinine of ≥30% (or decrease in eGFR of ≥10%) together with active urine sediment and an increase in glomerular hematuria of ≥10 red blood cells per high-power field, irrespective of proteinuria changes [1].

Damage accrual was defined as a ≥1-point increase in the Systemic Lupus International Collaborating Clinics/ American College of Rheumatology (SLICC/ACR) Damage Index (SDI) score [2] at any visit compared to the previous one.

*Statistical analysis*

Demographic, clinical and laboratory data are summarized using descriptive statistics. Continuous variables are expressed as means with standard deviations or medians with interquartile ranges, depending on data distribution. Categorical variables are expressed as frequencies and percentages. Extra-renal disease activity was characterized descriptively at visit level during follow-up.

The frequency and concurrence of active non-renal SLEDAI-2K domains were visualized using an UpSet plot. Patient-level activity profiles were summarized as the proportion of visits with each non-renal SLEDAI-2K component active, and displayed using hierarchical clustering.

**Supplementary Table S1.** Patient distribution across participating centers

| **Hospital** | **Department / Unit** | **Patients, n (%)** |
| --- | --- | --- |
| Attikon University Hospital | Rheumatology | 81 (48.2%) |
| University Hospital of Heraklion | Rheumatology | 20 (11.9%) |
| University Hospital of Ioannina | Rheumatology | 19 (11.3%) |
| G.Gennimatas General Hospital | Nephrology | 17 (10.1%) |
| University Hospital Centre Zagreb | Rheumatology | 13 (7.7%) |
| General Hospital Hippokration | Rheumatology | 11 (6.5%) |
| G.Gennimatas General Hospital | Rheumatology | 7 (4.2%) |

| **Latent Class** | **N visits** | **Mean Posterior Probability** |
| --- | --- | --- |
| Mucocutaneous/ Musculoskeletal (Class 1) | 315 | 0.956 |
| Serology/Mucocutaneous/ Musculoskeletal (Class 2) | 586 | 1.000 |
| Hypocomplementemia (Class 3) | 307 | 0.904 |
| **Entropy R² = 0.92** | | |

**Supplementary Table S2.** Latent class assignment quality metrics.

Classification quality was assessed using entropy R^2^ and mean posterior class assignment probabilities. Mean posterior probability reflects the average certainty of class assignment for visits assigned to each latent class via modal assignment. All three classes demonstrated high mean posterior probabilities (>0.90), indicating excellent assignment certainty and well-separated latent classes. Entropy R² provides an overall measure of class separation, with values >0.80 considered indicative of well-separated, clearly distinct latent classes.

**Supplementary Figure S1.** Non-renal SLEDAI-2K trajectories according to prior major organ manifestations.

Lines represent trajectories of mean non-renal SLEDAI-2K over follow-up among patients with and without prior major organ manifestations before lupus nephritis. Points indicate observed mean non-renal SLEDAI-2K values at each follow-up time point, and shaded bands represent 95% confidence intervals. In a visit-level GEE model accounting for repeated observations within patients and adjusted for follow-up time using a natural spline for month, prior major organ manifestation was associated with higher non-renal SLEDAI-2K during follow-up with a mean difference 0.69 points, 95% CI 0.08 to 1.31, p=0.026.

**Supplementary Figure S2.** Prevalence of extra-renal BILAG flares by domain during follow-up visits.

Grouped bar chart showing the percentage of follow-up visits with extra-renal BILAG flares in each domain (BILAG-A, BILAG-B and BILAG-A/B extra-renal flares). Bars represent the proportion of follow-up visits with extra-renal flare in each domain.

**Supplementary Figure S3**. Temporal trends in active extra-renal SLEDAI-2K domains following renal response in lupus nephritis.

Line plot showing the proportion of patients with active disease in each extra-renal SLEDAI-2K domain at each time point across 120 months of follow-up from first renal response. Each point represents the percentage of patients with a domain score >0 at that month. Only patients with at least one follow-up visit were included at each time point.

**Supplementary Figure S4.** Sensitivity analyses for the association of extra-renal disease activity with renal flares and damage accrual

Forest plot showing hazard ratios and 95% confidence intervals for associations of non-renal SLEDAI-2K, clinical non-renal SLEDAI-2K (excluding serologic components), and BILAG extra-renal flares with renal flares and damage accrual. All models used the Anderson-Gill counting process method to account for recurrent events, with robust variance estimation through patient clustering.

MMF and BEL models were adjusted for age, sex, type of initial renal response, baseline SDI, proliferative LN histology, SLE duration prior to LN, time-varying glucocorticoid dose, and time-varying MMF or BEL use, respectively. The CRR-only model was restricted to complete renal responders and adjusted for age and sex. The ≥12-month follow-up model was restricted to patients with at least 12 months of follow-up and adjusted as the main multivariable models.

**Supplementary Figure S5.** Latent class model selection for extra-renal disease activity patterns.

Line plot showing the Bayesian Information Criterion (BIC) for latent class analysis (LCA) models, restricted to visits with at least one active extra-renal SLEDAI-2K component. LCA was applied to binary indicators of individual non-renal SLEDAI-2K components at each visit. Lower BIC values indicate better model fit penalised for model complexity. The red point denotes the BIC-optimal solution at k = 3 classes, which was selected as the best balance between parsimony and fit. Models were fitted using 10 random starts to avoid local maxima.

**Supplementary Figure S6.** Evolution of extra-renal disease activity patterns during follow-up.

Stacked area plot showing the proportion of follow-up visits assigned to each disease state at each time point across 120 months following first renal response. Disease states were derived from a latent class analysis with active visits being assigned to one of three latent classes: Mucocutaneous/ Musculoskeletal (Class 1), Serology/Mucocutaneous/ Musculoskeletal (Class 2), and Hypocomplementemia (Class 3), based on modal posterior probability assignment. The dashed vertical line indicates the median follow-up of 48 months; estimates beyond this point are based on a decreasing number of patients and should be interpreted with caution.

**Supplementary Figure S7.** Distribution of maintenance treatment across extra-renal disease activity patterns.

Bar chart showing the percentage of follow-up visits in which each maintenance therapy was used across the disease activity states derived from the LCA. Bars represent the proportion of visits receiving mycophenolate mofetil (MMF), azathioprine (AZA), belimumab (BEL), and calcineurin inhibitors (CNI).

**Supplementary Figure S8.** Sensitivity analyses for the association of extra-renal disease activity classes with renal flares and organ damage accrual.

Forest plot with hazard ratios and 95% confidence intervals for the association between extra-renal disease state with renal flares and damage accrual. Disease states were derived from latent class analysis where active visits were assigned to one of three latent classes (Mucocutaneous/Musculoskeletal (Class 1), Serology/Mucocutaneous/ Musculoskeletal (Class 2), and Hypocomplementemia (Class 3). All models used the Anderson-Gill counting process method to account for recurrent events, with robust variance estimation through clustering by patient. The extra-renal quiescent state served as the reference category.

MMF and BEL models were adjusted for age, sex, type of initial renal response, baseline SDI, proliferative LN histology, SLE duration prior to LN, time-varying glucocorticoid dose, and time-varying MMF or BEL use, respectively. The CRR-only model was restricted to complete renal responders and adjusted for age and sex. The ≥12-month follow-up model was restricted to patients with at least 12 months of follow-up and adjusted as the main multivariable models.

**Supplementary Figure S9.** Incidence rates of renal and non-renal damage accrual across extra-renal disease activity states.

Bar plot showing incidence rates of renal and non-renal damage accrual, expressed per 100 person-years, according to extra-renal disease activity state. Damage accrual events were classified as renal or non-renal based on the SDI domain contributing to the ≥1-point increase. Horizontal error bars represent Poisson 95% confidence intervals, and labels indicate the number of damage accrual events in each category.

**References**

1	Bertsias GK, Tektonidou M, Amoura Z, *et al.* Joint European League Against Rheumatism and European Renal Association–European Dialysis and Transplant Association (EULAR/ERA-EDTA) recommendations for the management of adult and paediatric lupus nephritis. *Annals of the Rheumatic Diseases*. 2012;71:1771–82. doi: 10.1136/annrheumdis-2012-201940

2	Gladman D, Ginzler E, Goldsmith C, *et al.* The development and initial validation of the systemic lupus international collaborating clinics/American college of rheumatology damage index for systemic lupus erythematosus. *Arthritis & Rheumatism*. 1996;39:363–9. doi: 10.1002/art.1780390303
